# Assessing the assessments: Taking stock of learning outcomes data in India

**DOI:** 10.1016/j.ijedudev.2021.102409

**Published:** 2021-07

**Authors:** Doug Johnson, Andres Parrado

**Affiliations:** a Independent Researcher; b MIT, Economics Department, United States

**Keywords:** Learning outcome data, ASER, NAS, Variance decomposition

## Abstract

Data on learning outcomes is essential for tracking progress in achieving education goals, understanding what education policies work (and don’t work), and holding public officials accountable. We assess the accuracy and reliability of India’s two nationally representative surveys on learning outcomes, ASER and NAS, so that users of these datasets may better understand when, and for what purposes, these two datasets can reasonably be used. After restricting our sample to maximize comparability between the two datasets, we find that NAS state averages are significantly higher than ASER state averages and averages from an independently conducted and nationally representative survey (IHDS). In addition, state rankings based on NAS data display almost no correlation with state rankings based on ASER, IHDS, or net state domestic product per capita. We conclude that NAS state averages are likely artificially high and contain little information about states’ relative performance. The presence of severe bias in the NAS data suggests that this data should be used carefully or not at all for comparisons between states, constructing learning profiles, or any other purpose. We then analyze the internal reliability of ASER data using variance decomposition methods. We find that while ASER data is mostly reliable for comparing state averages, it is less reliable for looking at district averages, or changes in district and state averages over time. We conclude that analysts may use ASER data with confidence for comparisons between states in a single year, constructing learning profiles, and assessing learning inequality but should exercise caution when comparing changes in state scores and avoid using ASER district-level data.

## Highlights

• We assess the reliability of India’s two main sources of representative data on learning outcomes: the ASER and NAS tests.
• We find that NAS state averages are unrealistically high and contain little information about relative state performance.
• ASER data is reliable for comparing state averages but less so for looking at changes in state averages or district averages.
•Analysts should refrain from using NAS to make comparisons across districts/states or assess improvements over time.

## 1 Introduction

India is facing a learning crisis. In 2018, nearly half of all rural students in grade five couldn’t read a grade two text and two thirds couldn’t perform simple division (Pratham, 2018). While opinions vary on how best to address the learning crisis, there is widespread agreement that data on learning outcomes will be key to finding solutions (Figlio et al., 2016). The World Bank, in the 2018 World Development Report on education, urges countries “to take learning seriously, start by measuring it” (Filmer et al., 2018). Similarly, Pritchett (2015) argues that data on learning outcomes is key to ensuring that education systems are “coherent” for learning outcomes – that is, that the elements of the system are aligned around the objective of improving learning outcomes. As Pritchett points out, without data on whether children are achieving learning objectives, it is difficult to ensure that the system is sufficiently aligned with the ultimate goal of learning. A large body of evidence shows that in many developing countries, education systems are *not* coherent for learning outcomes: curricula are over-ambitious, thus leaving many children behind; exit exams are not aligned with curricular content; and teachers and administrators are rarely held accountable for achieving learning outcomes (Pritchett and Beatty, 2015; Atuhurra and Kaffenberger, 2020; Pritchett and Murgai, 2007).

In this paper, we take stock of India’s data on learning outcomes. In particular, we assess the accuracy and precision of data from India’s two nationally representative learning outcomes surveys: the Annual State of Education Report (ASER) basic survey, conducted by the independently run ASER Centre, and the National Achievement Survey (NAS), conducted by the central government with the help of the states. Our objective in assessing the accuracy and precision of these datasets is to provide guidance on when, and for what purposes, these datasets can reasonably be used.

The ASER basic survey, conducted every year from 2005 to 2014 and every other year starting in 2014, is representative of all rural households and seeks to measure whether children have attained basic foundational literacy and numeracy. To our knowledge, ASER was the first nationally representative survey of learning outcomes and has played a pivotal role in raising awareness of India’s low learning levels (Bridgespan, 2018).

The NAS (in its current, expanded format) has only been conducted once, in 2017, but the central government plans to conduct it regularly.^1^ NAS is administered in school to children in grades 3, 5, and 8^2^ and seeks to measure whether students have achieved grade-level learning objectives. In addition to these two sources of data on learning outcomes based on sample surveys, other potential sources of data on learning outcomes include state summative assessments and results from the board exams, administered at the end of classes 10 and 12. We do not consider summative assessments as these vary widely by state and are not made available to the public. Similarly, we do not consider board exams as a substantial portion of students do not complete grade 10 and state boards vary widely.

We first compare NAS and ASER data to each other and to a third source of data on learning outcomes, the India Human Development Survey (IHDS) (Desai and Vanneman, 2015). ASER and IHDS use a virtually identical assessment tool and a similar sampling strategy. By contrast, NAS uses a different assessment tool and sampling strategy. To ensure comparability across datasets, we focus on students and schools which are included in all datasets (rural class 3 students in government schools) and learning outcomes which are most similar across the datasets (reading outcomes). We caution that NAS does not publicly release the questions it uses and thus we are unable to completely ensure that the NAS and ASER assessments measure the same student skills.

After restricting the dataset samples, we find that ASER and IHDS state averages are very similar to each other. This is unsurprising given that the two datasets use the same tool and a similar sampling strategy, but nevertheless provides reassurance in the accuracy of ASER state averages. By contrast, we find that NAS state averages are significantly higher than both ASER and IHDS averages. In addition, state rankings based on NAS data display almost no correlation with state rankings based on ASER, IHDS, or Net State Domestic Product (NSDP). Given the high documented correlation between wealth and learning levels both across countries and within countries (for example Barro and Lee (2013) and Hanushek and Woessmann (2010)), the large role states play in delivering education, and the large variation in income across Indian states, this absence of correlation is surprising.

We show that the size of these discrepancies is larger than can be reasonably explained by differences in the latent reading ability being tested. We further provide suggestive evidence that voluntary student absence on NAS exam data is unlikely to be a major source of these discrepancies. We conclude that NAS state averages are likely artificially high and contain little information about states’ relative performance.

We next assess the internal reliability of ASER data. The ASER reading and math assessments have been analyzed through comparisons to other widely used tools like the Early Grade Reading Assessment (EGRA).^3^ These comparisons found ASER to be reliable and valid and the sample size for the ASER survey is large enough to ensure reasonable precision (Vagh, 2012). Yet, there are two reasons to suspect that there may be significant non-sampling errors in ASER data. First, ASER is implemented through the assistance of partner organizations which in turn often use volunteer surveyors with relatively little experience. Second, to sample households within villages, ASER uses the “right-hand rule,” in which surveyors walk around the village selecting every Xth household rather than the more accurate (but costly) household listing method. These are not criticisms of the ASER survey – without these cost-saving measures the survey would likely be prohibitively expensive. But these procedures do raise the risk of measurement error. And while all ASER enumerators undergo standardized training, even slight differences in survey administration by partner organizations may lead to large increases in the variance of district or state averages.

ASER data, unlike NAS, is available for several years and comparable across time periods, which allows us to use time-series techniques to assess the reliability of ASER over time. To do so, we use two approaches developed by Kane and Staiger (2002) for decomposing the variance of a time series into persistent and transitory components. We then further decompose variance arising from the transitory component into variance arising from sampling and variance arising from other sources. While we cannot further distinguish between transitory non-sampling variance arising due to surveying (such as partner fixed effects) or other sources (such as a temporary increase in learning outcomes), we show that learning level differences between cohorts are unlikely to be a cause of transitory changes in test scores. Additionally, we also provide qualitative arguments for why true changes in learning outcomes are unlikely to be the source of transitory changes in scores.

We apply these variance decomposition methods to state-level ASER data on the proportion of rural class 3 children who can read a standard 2 level text and the proportion who can perform simple subtraction. We also apply these methods to district-level data on the proportion of class 3, 4, and 5 students who can read a standard 1 level text and the proportion who can perform simple subtraction. We look at both a) learning levels, i.e. the average score for a state or district in a given year, and b) learning changes, i.e. the change in the average score for a state or district from one year to the next. We find that a relatively small portion (5–9 %) of the overall variance in state learning levels is due to transitory effects. By contrast, a substantial portion (between one third and one half) of the variance in changes in state scores and the variance in district learning levels are due to transitory effects. Variance in changes in district scores is nearly entirely (>75 %) due to transitory effects. Across subjects, aggregation levels, and levels vs changes, sampling error appears to account for a small portion of the variance.

Our findings have implications for how these two datasets are used. National learning outcomes data is most commonly used to compare the relative performance of states or districts and to assess progress within states or districts over time. One example is the School Education Quality Index (SEQI) created by India’s federal think tank, the NITI Aayog. The purpose of the SEQI is to “drive policy reform that will improve the quality of school education” and is based to a large extent on NAS data.^4^ Our findings suggest that NAS data should not be used these purposes. By contrast, we find that ASER data is more or less reliable for static comparisons of state performance, but care should be taken when using ASER to compare districts or state progress from one round to the next. Taking changes in average reading test scores at the state level as an example, approximately 40 % of the variance in the changes is due to transitory effects. This implies that if we attempt to identify the top 25 % of states in terms of reading gains, a third of the states identified would not actually be in the top 25 %.

In addition to comparing states and districts, national learning outcomes data are also increasingly used to estimate “learning profiles” and to assess inequality in education systems. Learning profiles depict the progress in average learning outcomes by age or grade in an education system to better understand where an education system succeeds and where it falls short (Kaffenberger, 2019). Assessments of learning equality compare average learning outcomes between groups, such as wealth quintiles, or analyze the overall distribution of learning in a population (Akmal and Pritchett, 2019; Rodriguez-Segura et al., 2020). In light of these novel ways to characterize national learning outcome differences, it is paramount to better understand the reliability of the underlying data.

We caution that do not directly assess the reliability of national averages by age, grade, or household characteristic from ASER or NAS data. Nevertheless, the presence of severe unexplained bias in the NAS data suggests that the data should probably not be used for the purposes of constructing learning profiles or assessing learning inequality. By contrast, we believe that analysts may use ASER for these purposes with confidence. The most likely source of measurement error in ASER data is differences in implementation of the survey by district partners. If so, these differences would not cause substantial noise in overall age or grade averages (due to the roughly equal distribution of ages and grades across districts) and only a small amount of noise for averages by household characteristics. This noise is likely to be small compared to the bias in inequality measures due the design of the ASER test or imperfections in determining wealth quintile. For example, Bond and Lang (2013) show that changes in test design or score construction can lead to significant differences in estimates of the black-white score gap in the US. Similarly, imprecision in the assignment of households to wealth quintiles based on principal components analysis or other methods likely leads to attenuated estimates of learning differences between wealth quintiles (Kolenikov and Angeles, 2009).

This paper also contributes to the growing literature on the overall quality of learning outcomes data. Chay et al. (2005) show that transitory noise and mean reversion in test scores lead to upwardly biased estimates of the impact of a government-sponsored education program in Chile. Similarly, Mizala et al. (2007) use panel data from Chile’s national standardized testing system to show that school rankings based on test scores are either uninformative beyond simply capturing socio-economic status or deeply noisy and volatile. Kane et al. (2002) and Kane and Staiger (2002) show that imprecision in test scores can severely affect school accountability systems which rely on these measures. In a study that is more closely related to our work, Singh (2020) finds that paper-based assessments proctored by teachers exaggerate student achievement in the Indian state of Andhra Pradesh. Singh (2020) points out that many studies in education assume that student test scores are reliable and thus can be leveraged to estimate the impact of education interventions. We show that in the case of India, reliability varies substantially across nationally representative datasets on educational outcomes, which presents an enormous challenge for using these datasets (particularly NAS) for subsequent research work.

The rest of this paper proceeds as follows. Section 1 provides a brief overview of NAS, ASER, and IHDS. Section 2 describes the overall approach to comparing these three sources of learning data. Section 3 presents the main results of this comparison. Section 4 presents the analysis on the internal reliability of ASER data. Finally, Section 5 concludes.

## 2 Sources of learning outcomes data

We first provide a brief background on each of the three learning outcomes surveys: ASER, NAS, and IHDS. In particular, we summarize each survey’s sampling strategy, frequency, test instrument, and implementation.

### 2.1 Annual State of Education Report (ASER) survey

The ASER basic survey is a nationally representative survey that seeks to assess rural Indian children’s basic literacy and numeracy. It was conducted every year in its first years but it is currently conducted every other year. The ASER basic survey uses a two-stage sampling strategy to select a representative sample of all rural households. In the first stage, 30 villages are selected using probability proportional to size without replacement (where size is defined as the number of households from the census) in each rural district in the country. Urban districts are excluded from the survey. The ASER basic survey employs a rotating panel of villages. Each year, 10 villages are replaced with new villages. In each village, 20 households are selected using the “right-hand rule,” a pseudo-random method for selecting households which does not require a full household listing.^5^

ASER surveyors collect data on school enrolment for all children ages 3–16 in selected households. In addition, ASER surveyors administer ASER reading and math assessments to all children ages 5−16. The ASER reading and math assessments are simple tools, conducted orally and one-on-one, designed to assess a child’s basic numeracy and literacy. The ASER reading assessment assigns each child one of five literacy levels: can’t identify letters, can identify letters but not words, can read words but not a paragraph, can read a short paragraph but not story, and can read a longer story (which corresponds to a standard 2 level text). Similarly, the ASER math assessment assigns each child one of five numeracy levels: can’t identify numbers 1–9, can identify numbers 1–9 but not 11–99, can perform two-digit subtraction but not 3 digit by 1 division, and can perform 3 digit by 1 division.

The entire ASER survey is implemented by a network of partner organizations and volunteers. In many districts, the ASER partner organization is the local District Institute of Educational Training (DIET). As noted below, NAS surveyors are recruited from candidates currently training to be teachers at DIETs.

### 2.2 National Achievement Survey (NAS)

The National Achievement Survey (NAS) is a large, school-based assessment of student learning conducted by the central government with the help of states. NAS has been conducted every year starting in 2001. In 2017 it was expanded to include children from grades 3, 5, and 8 at the same time (previous rounds typically assessed students from only one of these grades), the sample size was significantly increased so that results would be representative at the district level, and the assessment tool was modified to test student competencies. The central government also announced its intention to repeat this larger NAS in future rounds. For brevity’s sake, we, like most observers, refer to the 2017 NAS as the NAS though there have been several other rounds of this survey.

According to the NAS district report, 120,000 government and private-aided schools were selected from official lists for inclusion in NAS using probability proportional to size sampling. Within each school, up to 30 students per class in classes 3, 5, and 8 were randomly selected.^6^ NAS documentation does not specify how many schools were sampled per district or what measure of size (total number of students or total students in classes 3, 5, 8, and 10) was used. According to the NAS district report, a total of 2.2 million students were assessed in NAS making the NAS one of the largest sample surveys ever conducted.^7^

NAS collected a variety of data on schools and students and assessed all students’ language and math ability. In addition, NAS assessed class 3 and 5 students’ competency in environmental sciences and class 8 students’ competency in science and social science. The language and math assessments were designed to measure whether students had achieved learning outcomes as specified in the Right to Education Act (as amended in 2017). NAS does not make public the test questions it uses. Unlike the ASER assessment, the NAS assessment is a paper and pencil self-administered assessment. NAS was designed and supervised by the National Council of Educational Research and Training and implemented by states. Field investigators were selected from among candidates currently training to be government teachers at DIETs to ensure no conflict of interest.

### 2.3 India Human Development Survey (IHDS)

The India Human Development Survey (IHDS) is a large, panel survey representative of all households in India. We use only the second round of IHDS which was conducted in 2011−12. Households for this survey were selected using a two-stage sampling strategy.^8^

IHDS collected data on a range of subjects such as consumption expenditure, employment, and household assets. With respect to education, IHDS collected data on current enrolment, highest grade completed, and other education related variables for all household members. In addition, IHDS orally administered a learning assessment based on the ASER assessment tool to all children ages 8−11.

## 3 Comparison of ASER, NAS, and IHDS

Direct comparisons of overall results from IHDS, ASER and NAS are not valid. As Table 1 shows, the surveys are representative of different populations and NAS uses a different tool to assess learning outcomes. NAS gathers data on whether children attending government or private aided schools in grades 3, 5, and 8 have achieved learning objectives appropriate to their grade level in reading and math. ASER and IHDS gather data on whether rural children of ages 5–16 are able to read up to a standard 2 level text and whether they are able to perform math up to division. To facilitate comparison between the three different datasets, we restrict the sample of each of the datasets in several ways to ensure that the final three restricted datasets are as similar as possible.

**Table 1:** Summary of Learning Outcome Surveys.

	ASER basic	NAS	IHDS
Population for which results are representative	Rural children ages 5−16	Students attending government and private aided schools (and present on day of exam) in classes 3, 5, and 8	Children ages 8−11
Approximate sample size	320,000	2,200,000	11,693
Learning outcomes data collected	Basic literacy and numeracy; assessment tool administered orally and one-on-one	Math and language competency as defined by official learning objectives; student self-administered paper and pencil test	Identical to ASER
Other data collected	School enrolment	School infrastructure	Rich set of household information such as employment, expenditure, etc.
Field staff	Partner organizations and volunteers	State education officials and teacher candidates	Full-time trained survey team

First, we restrict the NAS and IHDS samples to students from rural areas as ASER is only administered in rural areas. Second, we restrict the three samples to ensure similarity in the types of schools covered. NAS is only administered in government and private aided schools. Unfortunately, we are not able to distinguish between students attending private and private aided schools in the ASER dataset, so we restrict the sample to students attending government schools. We include both government and private aided schools in the IHDS sample.

Third, we restrict our focus to NAS class 3 language outcomes and the proportion of class 3^9^ students who attained the highest reading level on the ASER assessment. To achieve the highest level on the ASER reading assessment a child must be able to read a standard 2 level text with 3 or fewer mistakes and should not read the text “haltingly” (though they may read slowly). This reading level appears similar, though more basic, than the two language learning outcomes measured by NAS for class 3 students: “reads printed scripts on the classroom walls: poems, posters, charts etc.” and “reads small texts with comprehension i.e., identify main ideas, details, sequence and draws conclusions.”^10^ The first NAS class 3 language learning outcome does not specify what level of text the classroom materials are at but presumably they are at a class 3 level. The ASER assessment and the second NAS class 3 language learning outcome differ slightly in that the ASER assessment does not directly measure student comprehension. A comparison of the ASER tool with other reading assessments which more directly measure student reading comprehension shows that achieving the highest level on the ASER assessment is strongly associated with being able to read and comprehend a grade 2 level text (Vagh, 2012). We caution that we do not know how well that the NAS assessment actually measures these learning outcomes. As stated above, NAS has not publicly released the assessment items used or details of any validation exercise. However, given that NAS aims to assess reading ability at class 3 level, we can infer that the NAS exam is nominally more demanding than ASER. Therefore, we should expect NAS scores to be lower, not higher, than the ASER scores in the most comparable sample.

We do not use data on math as it is more difficult to compare the NAS class 3 math learning outcomes to ASER math levels. Similarly, we do not use data for higher grades as the highest level of competency tested by the ASER assessment is well below official grade level competency for these grades. Restricting attention to class 3 students and the language portion of the ASER and NAS assessments helps ensure that the two assessments are measuring similar student skills. Yet without access to the NAS assessment tool, it is impossible to know for certain whether the NAS language assessment and the ASER reading assessment measure similar student skills.

Finally, we use data from the most recent ASER (conducted in 2018), which helps ensure comparability between NAS and ASER. We acknowledge that there is a substantial time difference between IHDS, conducted in 2011/12, and the other two datasets. However, given that we seek to compare the correlation between ASER and IHDS to the correlation between NAS and IHDS, this time difference does not pose a problem. Table 2 shows a summary of the restricted samples.

**Table 2:** Summary of Restricted Samples Used for Comparison.

	ASER basic	NAS	IHDS
Year of survey	2018	2017	2011−12
Grades	3	3	2−4
Learning outcomes	Ability to read grade 2 text	Ability to read printed scripts on the classroom walls and ability to read small texts with comprehension	Ability to read grade 2 text
Schools	Government	Government and private aided	Government and private aided
Rural/urban	Rural	Rural	Rural

We compare state averages for the three datasets. While it is theoretically possible to also compare ASER and NAS district averages (though not IHDS district averages due to limited sample size), our results for the comparison between state averages suggest that there would be little benefit to this comparison as the state averages diverge widely.

To better understand whether differences in the assessment tools may be driving differences in state averages between the datasets, we compare the correlation between state average NAS and ASER reading scores to the correlation between state average ASER reading and math scores (calculated by taking the correlation between scores in each year and averaging these correlations). We interpret the correlation between ASER reading and math scores as a crude lower bound of the correlation between ASER state averages and any other well-designed basic reading assessment administered to the same sample of children. While different assessments of basic reading may measure slightly different latent reading abilities, we would expect these latent basic reading abilities to be more highly correlated than basic reading and basic math. Further, previous research has shown that ASER performs well in measuring basic reading ability (Vagh 2012). If we find the correlation between ASER and NAS state averages is significantly lower than the correlation between ASER state reading and math, we can infer that either a) sampling or survey error is causing differences between the datasets or b) NAS does not accurately measure basic reading ability.

In addition to differences between the assessment tools, differences in the sampling strategy may also drive differences between the datasets. In particular, since NAS is administered at schools while ASER is administered at home, any state-level differences in the probability of low/high performing students showing up on NAS exam day would result in differences in the state averages for the two datasets. Of course, if the goal of the NAS survey is to obtain an accurate estimate of learning for all government school students we should still be concerned if we find that differences in test attendance are the main drivers of the dissimilarities in results. Nevertheless, understanding whether differential test attendance may be driving differences in results may be helpful in diagnosing any potential discrepancies between the datasets.

To test whether voluntary student absence on NAS exam day may be driving differences between the datasets we use self-reported data on school attendance from IHDS. Formally, we assume that the probability of attendance on NAS exam date is $\frac{25-DAYS_{i}}{25}$ where $DAYS_{i}$ is the self-reported number of days child i was absent from school in the previous month and calculate expected IHDS score taking into account probability of attendance. We caution that these results are only suggestive due to potential measurement error in this variable. In addition, this test only assesses the potential contribution of voluntary student absence. Teachers may have selectively encouraged certain students to stay at home on NAS exam day.

## 4 Results

**Fig. 1:**
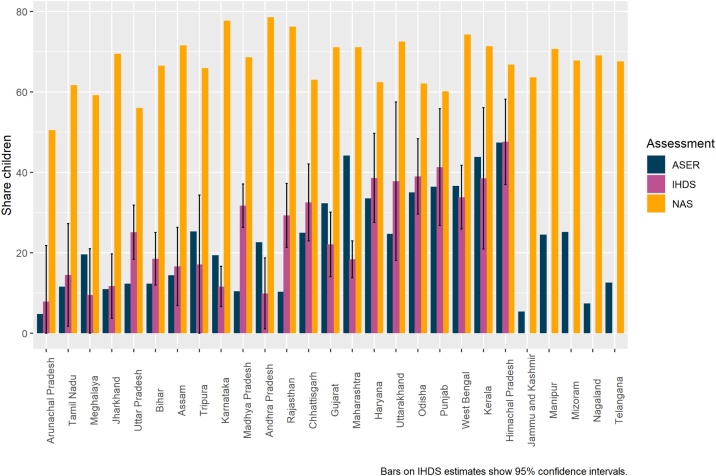
ASER, NAS, and IHDS Scores.

**Fig. 2:**
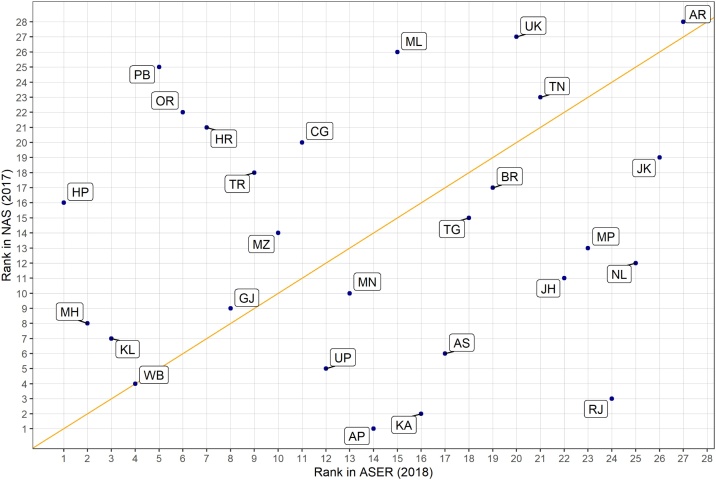
Correlation Between NAS and ASER State Ranks. Notes: ASER figures represent state rankings based on the share of rural class 3 students attending government schools who can read a class 2 text from ASER 2018. NAS figures represent state rankings based on the share of rural class 3 students attending government or private aided schools who have achieved the two class 3 language learning outcomes according to NAS 2017. For details of the NAS class 3 language learning outcomes see the methods section.

Notes: ASER figures represent the share of rural class 3 students attending government schools who can read a class 2 text from ASER 2018. IHDS figures represent the share of rural class 2, 3, and 4 students attending government or private aided schools who can read a class 2 text from IHDS 2011–12. Bars on IHDS scores show 95 % confidence intervals. (Without access to the raw data, we are unable to calculate confidence intervals for the other two datasets.) NAS figures represent the share of rural class 3 students attending government or private aided schools who have achieved the two class 3 language learning outcomes according to NAS 2017. For details of the NAS class 3 language learning outcomes see the methods section.

Fig. 1 plots class 3 average language scores for rural, government school students from ASER, NAS, and IHDS. IHDS values are missing from some states due to insufficient sample size. Fig. 2 plots the state rank from ASER on the x axis and the state rank from NAS on the y axis.

These figures show that IHDS and ASER state averages are very similar in size and that NAS state averages are much higher and not very correlated with either IHDS or ASER. A formal test for correlation confirms that IHDS and ASER are highly correlated (r = 0.62), and NAS is not at all correlated with IHDS (r = −0.03) and only modestly correlated with ASER (r = 0.19). For comparison, ASER grade 3 state average reading and math scores are highly correlated (r = 0.82) suggesting that differences in the aspect of reading being measured likely accounts for very little of this discrepancy.

In addition, comparing ASER and NAS to Net State Domestic Product (NSDP) reveals that ASER is substantially correlated with NSDP (r = 0.41) while NAS is only modestly correlated with NSDP (r = 0.05). All correlations are Pearson though Spearman gives similar results.

**Fig. 3:**
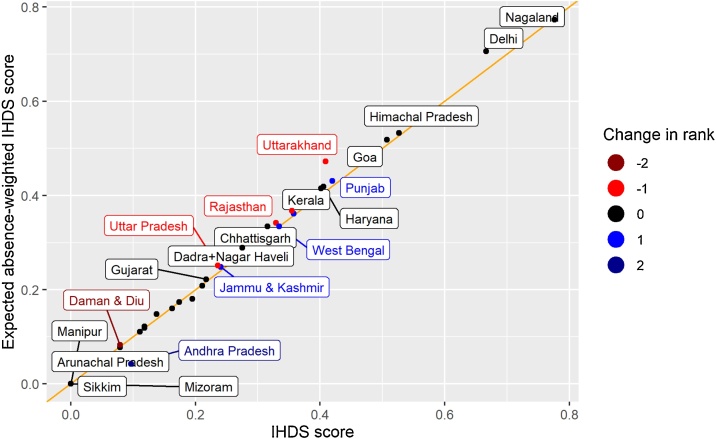
IHDS Score vs. Expected Absence Weighted Score. Notes: The x axis represents the share of rural class 2, 3, and 4 students attending government or private aided schools who can read a class 2 level text from IHDS 2011–12. The y axis is this same figure adjusted to account for student absence. See methods section above for details of adjustment procedure.

Fig. 3 plots state averages from IHDS taking absence into account (y axis) against state averages when absence is not considered (x axis). Most points in the figure lie slightly above the line of equality, revealing that students with higher rates of absence tend to have lower learning outcomes. However, the effect of these absences on overall scores is minimal. In only a few cases does taking absence into account shift relative ranking of the state. The cases where the relative rankings change are indicated by different colors.

## 5 Assessing ASER’s reliability

### 5.1 Overview of approach

In the next section, we estimate the internal reliability of ASER data by analyzing changes in ASER state and district scores over time. The objective of this analysis is to quantify the share of variance in ASER state or district scores (or changes in ASER scores) which is due to true differences between states or districts and the share which is due to measurement error. This allows users of ASER data to better understand for what purposes it is reasonable to use the data. For example, if the vast majority of the variance in state scores is due to true differences in state learning outcomes, then it is reasonable to use ASER scores to compare state learning outcomes. On the other hand, if the vast majority of the variance in district scores is due to measurement error, then ASER data should *not* be used to compare district learning outcomes.

While the similarity between ASER and IHDS data at the state level provides reassurance that ASER data does not contain large bias, it does not rule out the possibility that ASER contains significant measurement error. ASER’s reliance on partner organizations for surveying and the right-hand rule for sampling households generates potential for significant non-sampling errors. For example, partner organizations may differ slightly in how they supervise enumerators. We do not analyze the internal reliability of NAS data for two reasons. First, the analysis conducted in the previous section suggests that, even at the state level, there is large bias in NAS scores. Second, since NAS has only been conducted once in its present form, it is not possible to analyze changes in NAS scores over time.

We analyse the reliability of ASER data using two approaches adapted from Kane and Staiger (2002)’s analysis of average school test scores in the US. Kane and Staiger decompose the variance of school average test scores into persistent and transitory components and then further decompose the transitory component into sampling variance and non-sampling variance. Intuitively, this approach looks at whether changes in ASER scores from one round to the next are typically reversed. If changes in scores tend to “stick,” we can be relatively confident that measured changes reflects actual changes in underlying learning outcomes. On the other hand, if changes are typically reversed, we would suspect that measured changes often reflect measurement error.

The key assumption underlying this approach is that changes in measured scores that persist reflect true changes in learning outcomes while transitory changes are due to measurement error. This assumption would be violated if some true effects on learning outcomes are only transitory. True transitory effects on learning outcomes may arise from two main sources. First, some policy or intervention may cause a temporary increase/decrease in learning outcomes that is reversed in subsequent years. Second, one cohort of students may have higher/lower learning outcomes than cohorts above and below. If this is the case, the round in which those students are tested would show higher/lower learning outcomes.

We are unable to test whether temporary increases in learning outcomes are plausible based purely on ASER data, but we find it unlikely based on our understanding of education policy in India. Education policies are generally for multiple years and rarely are significant changes rolled back after a single year. By contrast, we are able to empirically test whether differences between cohorts are a likely source of transitory changes in ASER scores by looking at whether changes in grade 3 scores predict changes in grade 5 scores two years later.

### 5.2 Formal approach

We use two different methods to decompose variance into persistent and transitory components. The first method assumes that average test scores $y_{t}$ for a state or district at time t consist of a fixed component α, a persistent component $v_{t}$ which follows a random walk, and a transitory component $\varepsilon_{i}$ so that average test scores equal:

$$\begin{array}{c} y_{t}=\alpha +v_{t}+\in_{t}\\ where\;v_{t}=v_{t-1}+u_{t} \end{array}$$

We assume that the $u_{t}$ and $\varepsilon_{t}$ terms are independent of each other, mean zero, and i.i.d. Then, $Var(\Delta y_{t})=\sigma_{u}^{2}+2\sigma_{e}^{2}$ and the proportion of the overall variance of the changes in y arising due to the transitory shock can be estimated as:

$$\begin{array}{cc} -2*corr\left(\Delta y_{t},\Delta y_{t-1}\right) & =-2*corr\left(u_{t}+\in_{t}-\in_{t-1},u_{t-1}+\in_{t-1}-\in_{t-2}\right)\\ \; & =\frac{{2\sigma }_{e}^{2}}{\sigma_{u}^{2}+2\sigma_{e}^{2}} \end{array}$$

Similarly, we can also estimate the proportion of variance in levels (as opposed to changes) which are due to the transitory shock by rearranging the formula above to get:

$$\sigma_{e}^{2}=corr(\Delta y_{t},\Delta y_{t-1})\text{*}Var(\Delta y_{t})$$

The key assumptions behind this approach to decomposing variance are that the transitory component of the score, $\varepsilon_{t}$, is i.i.d. and the persistent component of the score, $v_{t}$, follows a random walk. These assumptions in turn imply that $u_{t}$ and $\varepsilon_{t}$ terms are not serially correlated. These are strong assumptions but conservative in the sense that most plausible violations of these assumptions will lead us to understate the share of variance due to the transitory component. For example, if states or districts often implement programs which result in not just one-off increases (decreases) in learning outcomes but multi-year increases (decreases) in learning outcomes the $u_{t}$ terms would exhibit positive serial correlation and our estimates of the proportion of variance due to transitory shocks will be downward biased. Similarly, if partner organizations collect data in the same areas for multiple years the $\varepsilon_{t}$ terms may be serially positive correlated which again would lead us to underestimate the share of variance due to transitory shocks. In Appendix A we compare this simple model with a dynamic value-added model that may be more familiar to education researchers.

In addition, we can partially test for serial correlation in the $u_{t}$ and $\varepsilon_{t}$ terms by looking at $corr(\Delta y_{t},\Delta y_{t-2})$. If the $u_{t}$ and $\varepsilon_{t}$ terms are not serially correlated, the correlation in current changes and twice lagged changes should be 0.^11^ We find that this holds approximately for district changes (correlation with double lag is 0.04 for reading and -0.04 for math) but not for the state changes (the correlation with double lag ranges from 0.1 to 0.18). Thus, for states we use a second method for decomposing variance into persistent and transitory components also developed by Kane and Staiger. We focus on results from this second method in the main results section but also present results from the first method in Table C1 in the Appendix C. Results from the first method do not alter our substantive results.

**Fig. 4:**
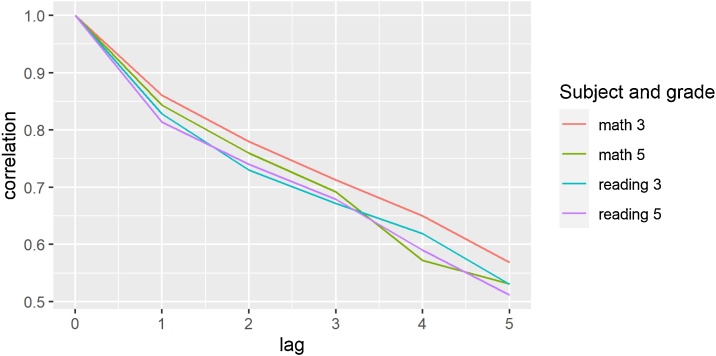
ASER Autocorrelation Decay. Notes: Each point represents the correlation between ASER state scores in year t and scores in year t-lag. Class 3 math scores are the share of students in each state able to do at least subtraction. Class 5 math scores are the share of students in each state able to do at least division. Class 3 reading scores are the share of students in each state able to read a class 1 level text. Class 5 reading scores are the share of students in each state able to read a class 2 level text.

The second method relies on the fact that if there is both a persistent component and a transitory component to scores, we would expect the correlation between current scores and the first lagged score to reflect both persistent and transitory shocks while the decay in autocorrelation for further lags would mainly reflect the persistent component. If this is the case, we would expect autocorrelation to fall quite a bit with the first lag and then exhibit relatively steady decay after that. We can assess this graphically by inspecting autocorrelation by lag number. The figure below shows the average autocorrelation between current state averages and previous state averages for lags up to five years. For both reading and math, the initial decrease in correlation (starting from 1) is larger than the subsequent decreases and subsequent decreases tend to be relatively stable (Fig. 4).

More formally, we may estimate the variance of the persistent component, $\sigma_{pers}^{2}$, by looking at the ratio of lag one autocorrelation over “persistent autocorrelation” which we define as the average decay in autocorrelation with further lags:

$$\begin{array}{l} \sigma_{pers}^{2}=1-\sigma_{\in }^{2}\\ \approx \frac{\sigma_{y}^{2}\rho }{\rho_{pers}}\\ where\;\rho_{pers}\approx K^{-1}\sum_{k=1}^{4}\frac{\rho_{k+1}}{\rho_{k}} \end{array}$$

And $\rho_{k}$ is the average correlation between current scores and the kth lag.

Once we have calculated $\sigma_{pers}^{2}$ and $\sigma_{sampling}^{2}$ (see below) we calculate the variance of non-sampling transitory effects as the residual, $\sigma_{other}^{2}=\sigma_{y}^{2}-\sigma_{pers}^{2}-\sigma_{sampling}^{2}$. For changes in state scores, we calculate the persistent effects as $\sigma_{pers,changes}^{2}=\sigma_{\Delta y}^{2}-2*\sigma_{other}^{2}-2*\sigma_{sampling}^{2}$.

For both methods, we decompose $\sigma_{\varepsilon }^{2}$ into variance arising from sampling and variance arising from other transitory effects using analytical estimates of sampling variance. ASER doesn’t publish standard errors and we don’t have access to the microdata, so we are unable to directly estimate the standard errors. However, we may estimate the sampling variance analytically using information on the ASER sampling strategy and the standard formula for sampling variance given a two-stage cluster sampling strategy. From Lohr (2019):

$$\sigma_{samp}^{2}=(1+(m-1)\text{*}ICC)\text{*}\frac{\sigma_{y}^{2}}{N}$$

Where $\sigma_{samp}^{2}$ is the sampling variance for variable y, m is the number of children per village for which data is collected, ICC is the intraclass correlation of ASER scores at the village level, N is the sample size, and $\sigma_{y}^{2}$ is the variance of the variable y in the population.

Within each district, ASER samples 30 villages and 20 households per village and thus m is 20 and N is 600. For a binary variable with prevalence .5 $\sigma_{y}^{2}=0.25$. Finally, we obtain an estimate for the ICC of 0.18 from IHDS data.

We compare this estimate with standard errors reported in a technical paper on ASER precision published by the ASER centre (Ramaswami and Wadhwa, 2010). Variance of estimates for districts reported in this paper are around 0.0016. The similarity between the two figures lends confidence to our estimates. We take as our final estimate of the variance of district estimates due to sampling as 0.0016, though other similar values don’t change our results substantially.

To calculate sampling variance at the state level, we divide this variance by the number of districts in the state and then take the average across states. While this approach is slightly crude, sampling variance at the state level is very small and thus unlikely to affect our results.

As previously mentioned, the critical assumption underlying this analysis is that transitory shocks to ASER scores are due to measurement error rather than actual changes in learning outcomes. One potential source of true transitory effects on learning outcomes is differences between cohorts. We may test whether cohort effects account for a substantial share of year-to-year changes by looking at whether grade 3 changes in scores anticipate grade 5 changes in scores. If differences between cohorts, rather than changes in learning gains between grade 3 and grade 5 or changes in measurement error, make up a large portion of overall changes in scores we would expect that a change in grade 3 scores from one year to the next would be reflected in a similar change in grade 5 scores two years later. Formally, we perform the following regression:

$${\Delta y}_{5,t}=\beta {\Delta y}_{3,t-2}+\varepsilon_{t}$$

Where ${\text{Δ}y}_{g,t}$ is the change in scores from year t-1 to year t holding grade constant. In Appendix B we show that if β≈0 then either changes in grade 5 scores are driven by changes in measurement error or else changes in true grade 5 learning levels are driven primarily by changes in learning gains between grade 3 and grade 5 rather than differences between cohorts.

The data we use for these analyses differs from that used above to compare ASER and NAS in several respects. First, state and district averages include all students, not just those attending government schools. Second, for districts we use the share of standard 3, 4, and 5 students who can at least read a standard 1 text and can at least perform subtraction. Our district data is from 2006 to 2011. ASER only publishes two variables for district averages (these variables and the share of standard 1 and 2 students who can recognize letters and who can recognize numbers). The variables chosen are closer to the variable used in the analysis above and also more likely to be stable over time due to the inclusion of 3 grade levels. For states, we use the a) share of class 3 children who can at least read a standard 1 text, b) the share of class 3 children who can do at least subtraction, c) the share of class 5 children who can read at least a standard 2 text, and d) the share of class 5 children who can perform simple division. Our state data is from 2006 to 2014. Again, our choice of variables is driven by availability of data. These are the only variables easily accessible for all years in our dataset.

### 5.3 ASER internal reliability results

**Fig. 5:**
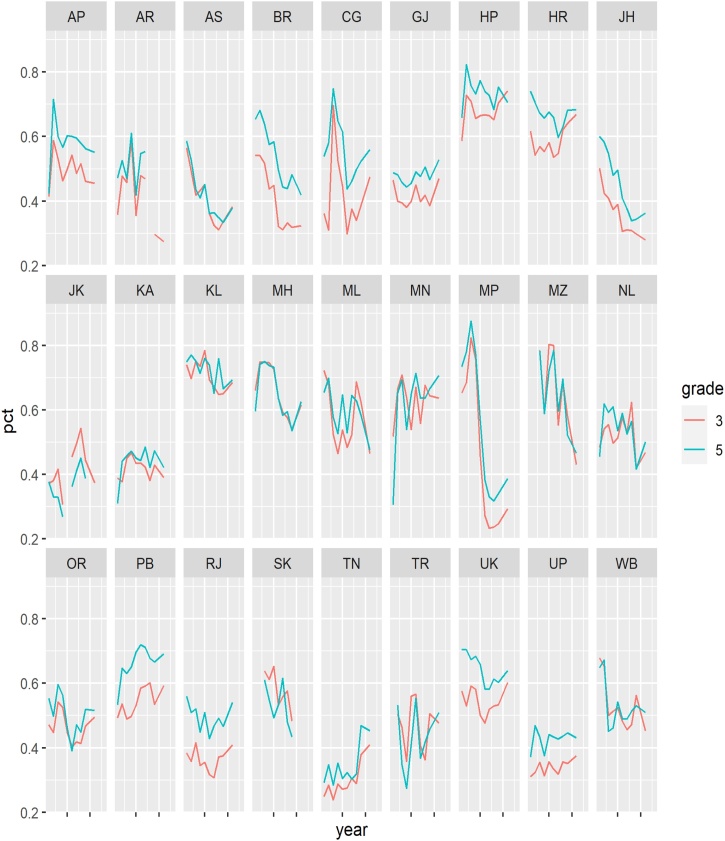
ASER Reading Levels Over Time by State. Notes: Class 3 reading scores are the share of students in each state able to read a class 1 level text. Class 5 reading scores are the share of students in each state able to read a class 2 level text.

**Fig. 6:**
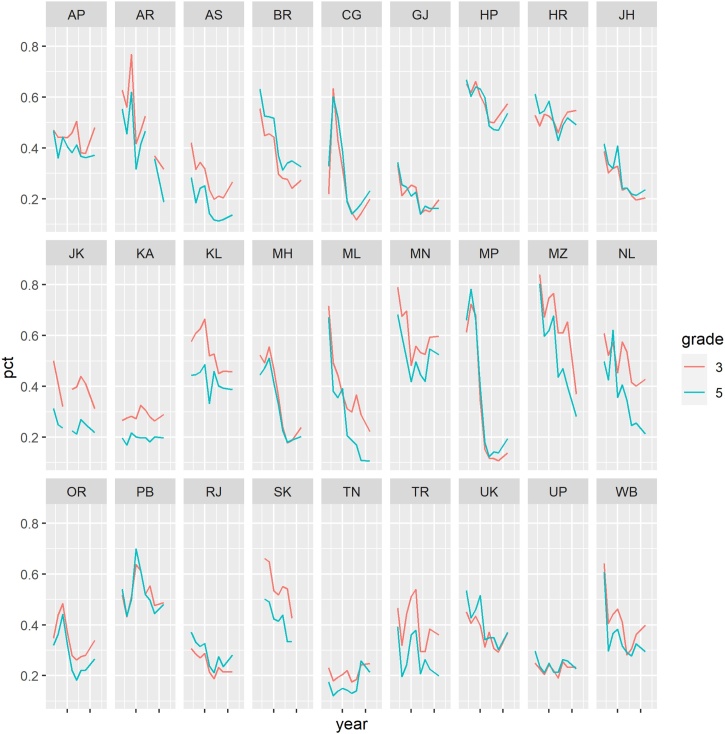
Math Levels Over Time by State. Notes: Class 3 math scores are the share of students in each state able to do at least subtraction. Class 5 math scores are the share of students in each state able to do at least division.

Fig. 5, Fig. 6 display ASER state reading and math scores for grade 3 and 5 over time. The figures show that even at the state level, ASER scores are quite “jumpy.” In addition, based on visual inspection it does not appear that grade 5 scores are influenced by lagged grade 3 scores.

**Fig. 7:**
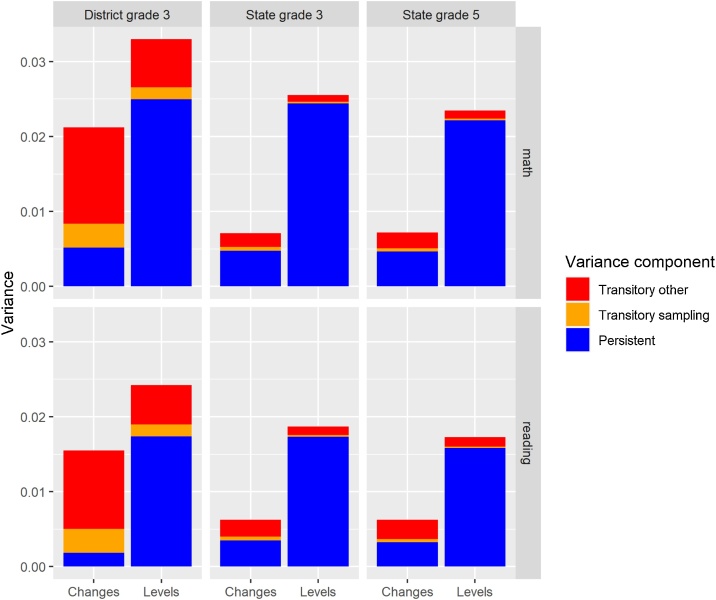
ASER Variance Decomposition. Notes: Figures represent variance in ASER scores at state and district level and for levels and changes due to a persistent component, sampling, and other transitory components. See methods section for details of estimation procedure.

Fig. 7 displays the breakup of variance into a persistent component, sampling, and non-sampling transitory effects. Table C1 in the Appendix C displays the same information but in numerical form and as shares of the total rather than absolute size.

For both reading and math, a large proportion (91 %–95 %) of the variance in state scores (i.e. levels) are due to persistent effects. The share of variance due to persistent effects is lower but still substantial for changes in state scores and district scores, ranging from 52 % for changes in state grade 5 reading scores to 76 % for grade 3 district math score levels. By contrast, the share of variance due to persistent effects is quite low for changes in district scores (24 % for math and 12 % for reading). For all subjects and aggregation levels and for both changes and levels, sampling variance makes up a relatively small share of overall variance and is much smaller than the variance due to other transitory effects.

Regressions of changes in class 5 state scores on twice lagged changes in grade 3 scores reveals that changes in grade 3 scores do not at all anticipate changes in grade 5 scores. The coefficient on twice lagged gains is -0.045 (std error = 0.069) for math and 0.036 (std error = .063) for reading. These results suggest that transitory effects are unlikely to be due to differences between cohorts.

If non-sampling transitory effects arise from survey error, these findings imply that comparisons between state levels based on ASER are relatively accurate but that comparisons between changes in state scores, districts, and changes in district scores will be less reliable. For example, taking grade 5 reading scores as an example, the variance decomposition implies that if we were to attempt to identify the top 25 % of states in terms of grade 5 reading scores, we would achieve roughly 75 % accuracy. By contrast, if we were to attempt to identify the top 25 % of states in terms of changes in 9 grade 5 reading scores, our accuracy would be only around 50 %.^12^

## 6 Conclusion

We find that in most states, the share of rural class 3 government and private aided school students who achieved grade level language competency on the 2017 NAS is much higher than the share of rural class 3 government school students who were able to read a grade 2 level text according to the 2018 ASER survey. We further find that NAS state rankings display almost no correlation with state rankings based on ASER and IHDS or net state domestic product per capita. We conclude that NAS state averages are likely artificially high and contain little information about states’ relative performance. We note, however, that NAS state rankings are calculated based on the share of rural class 3 government and private aided school students who achieved grade level language competency. Meanwhile, ASER and IHDS calculate state ranking based on the share of rural class 3 government school students who can read a grade 2 level text. Additionally, based on an analysis of internal reliability, we find that ASER data is mostly reliable for comparing state averages but less reliable for looking at changes in in state averages, district averages, or changes in district averages.

Our findings have broad implications for how the existing data is used as well as for potential future data collection efforts. Our results for NAS suggest that NAS state averages (not to mention district results) should be used with extreme care, if at all. This result is particularly important in light of the various policy recommendations underway based on NAS data, like India’s State Education Quality Index. On the other hand, our analysis suggests that ASER is indeed a reliable guide for comparing state progress in basic literacy and numeracy, but that care should be taken when comparing changes in indicators across states. Comparisons of changes in two states should be considered suggestive if the difference in their changes is small and rankings based on changes should be considered indicative. Researchers seeking to use ASER to estimate the impact of a policy may consider techniques which allow for error such as the methods described in Griliches and Hausman (1986).

Taken together, these findings reveal a need for more precise data on learning outcomes in India. Data on learning outcomes for all children (those attending government and private schools in both rural and urban areas) with small standard errors at the state level would allow policymakers and the public to more accurately track progress in meetings the goals of the soon to be launched National Foundational Literacy and Numeracy Mission and researchers to more precisely estimate the impacts of education programs.

Our findings, along with other research in this space, also suggests ways to fill (or not fill) this gap. First, the disappointing results for the NAS data provide further evidence that collecting accurate data on learning outcomes, especially using assessments administered in schools, is exceptionally hard. Analysis of NAS training and guidance documents shows that much thought and care went into this exercise. For example, the method for randomly selecting students in classrooms, in our opinion, carefully balances the need for random selection with the need for practical feasibility. Our findings is in line with evidence from Madhya Pradesh where Singh shows that scores on a set of assessments administered in schools were artificially inflated even though there were little to no consequences for having high or low scores (though he finds that the assessments contained useful information about relative student/school performance) (Singh, 2020).

Second, we show that sampling variance accounts for a relatively small share (between one fourth and one ninth) of uncertainty in ASER state level estimates. This suggests that a survey with a smaller sample size, but also less non-sampling variance, could achieve similar levels of precision. For example, if a learning outcomes survey were to achieve zero non-sampling error it could attain ASER-levels of precision with only 1/16 to 1/81 the sample size (where we reduce sample size by reducing the number of villages rather than reducing students per village).

Taken together, this suggests that a smaller, household-based survey of learning outcomes using a tool similar to ASER but with more direct oversight and use of a full household listing for sampling may be a promising approach for collecting learning outcomes data. One option would be to add on an ASER-like tool to one of the many large household surveys regularly conducted in India. Use of the ASER tool in the IHDS shows that this could be done for little marginal cost or hassle. In addition, the rich set of additional household variables would allow for increased precision of district and state learning outcomes through small area estimation and advanced imputation for missing assessment scores.

## CRediT authorship contribution statement

**Doug Johnson:** Conceptualization, Methodology, Software, Formal analysis, Resources, Data curation, Writing - original draft, Writing - review & editing, Visualization. **Andres Parrado:** Conceptualization, Software, Resources, Data curation, Writing - original draft, Writing - review & editing, Visualization.

## Figures and Tables

**Table C1:** Detailed ASER Variance Decomposition Results.

State/District	Change/Levels	Subject	Grade	Share persistent	Share sampling	Share other	Share persistent (alt method)	Share sampling (alt method)	Share other (alt method)
District	Changes	Math	3	24 %	15 %	61 %	NA	NA	NA
District	Changes	Reading	3	12 %	21 %	67 %	NA	NA	NA
District	Levels	Math	3	76 %	5 %	19 %	NA	NA	NA
District	Levels	Reading	3	72 %	7 %	22 %	NA	NA	NA
State	Changes	Math	3	67 %	7 %	26 %	67 %	7 %	26 %
State	Changes	Math	5	65 %	6 %	30 %	45 %	6 %	50 %
State	Changes	Reading	3	56 %	8 %	36 %	47 %	8 %	44 %
State	Changes	Reading	5	52 %	7 %	41 %	42 %	7 %	52 %
State	Levels	Math	3	95 %	1 %	4 %	95 %	1 %	4 %
State	Levels	Math	5	95 %	1 %	5 %	92 %	1 %	8 %
State	Levels	Reading	3	93 %	1 %	6 %	91 %	1 %	7 %
State	Levels	Reading	5	91 %	1 %	7 %	89 %	1 %	9 %

*Notes*: Figures represent share of variance in ASER scores at state and district level and for levels and changes due to a persistent component, sampling, and other transitory components. See methods section for details of estimation procedure.

## Appendix A

When analyzing student-level panel data, education researchers often utilize a value-added model for learning outcomes along the lines of:

$$y_{t}=\beta y_{t-1}+\gamma X_{t}+u_{t}$$

See Todd and Wolpin (2003) for details. We could, in theory, model state and district level results using a similar value-added model. To do this, we first assume that state/district scores are the sum of the true underlying score, $y_{t}^{*}$, and a centered i.i.d. measurement error, $\varepsilon_{t}$. (We avoid the terms “persistent” and “transitory” here for reasons that will be clear momentarily.) Then we may model the true underling score using a value-added model:

$$y_{t}^{\text{*}}=\beta y_{t-1}^{\text{*}}+\eta +u_{t}$$

Where $u_{t}$ and $\varepsilon_{t}$, as before, are independent of each other, mean zero, and i.i.d. The key difference between this model and the simple AR(1) model we use in the main text is that this model implies mean reversion of scores to η1-β over time (and thus we cannot label $y_{t}^{*}$ the “persistent” component) while the AR(1) model implies a random walk.

While useful for modelling student-level learning outcomes, such a value-added model is less appropriate for modelling mean scores of a region over time. At the student-level, the assumption of mean reversion is a reasonable one. Research on the persistence of learning shows that students lose a large amount of previous learning gains without further inputs (Andrabi et al., 2011). At a regional level, it is less clear what would lead to imperfect persistence of mean learning gains. Indeed, at the country level at least, learning outcomes do not appear to be converging (Pritchett, 2013).

Two further advantages of the simple AR(1) model is the ease with which it can be used to estimate the share of variance from persistent vs transitory components and the fact that it is conservative in the sense that most plausible deviations from the assumptions will lead to under-estimation of the share of variance due to transitory components. Estimation of the share of variance due to $y_{t}^{*}$ versus $\varepsilon_{t}$ in the model above is possible using GMM but complicated and reliant on strong and opaque assumptions. To estimate $\sigma_{\varepsilon }^{2}$, we may use the moment conditions

$$\begin{array}{l} E\left[y_{t}-\beta y_{t-1}-\eta \right]=0\\ E\left[y_{t-2}\left(y_{t}-\beta y_{t-1}-\eta \right)\right]=0\\ E\left[\left(\Delta y_{t}-\beta \Delta y_{t-1}\right)\left(\Delta y_{t-2}-\beta \Delta y_{t-3}\right)\right]=-\beta \sigma_{\varepsilon }^{2} \end{array}$$

While parameters of this model are identified with t≥3, the assumptions behind estimation are difficult to interpret in practical terms and plausible violations of the assumptions could lead to either over or under-estimation of $\sigma_{\varepsilon }^{2}$.

## Appendix B

Define true learning levels in grade g at the end^13^ of year t as $c_{g,t}$ and true learning gain in grade g in year t as $l_{g,t}$. If we assume no dropouts and that all students advance a grade each year, then:

$$c_{g,t}=c_{g-1,t-1}+l_{g,t}$$

And

$$c_{5,t}=c_{3,t-2}+l_{4,t-1}+l_{5,t}$$

We do not observe true learning levels. Instead, we observe measured learning levels $y_{g,t}$ which are equal to true learning levels plus a noise term $\varepsilon_{g,t}$.

Note that there is no clear mapping between the decomposition of scores into persistent and transitory components in this new formulation (aside from the fact that the noise term is clearly transitory): changes in c or l may be persistent or transitory. Our goal is not to show that cohort effects are unlikely to be transitory but rather to test for cohort effects at all. If we can rule out cohort effects, we may conclude that transitory effects are unlikely to be due to cohort effects.

Define ${\text{Δ}c}_{g,t}=c_{g,t}-c_{g,t-1};\;{\text{Δ}l}_{g,t}=l_{g,t}-l_{g,t-1};\;{\text{Δ}y}_{g,t}=y_{g,t}-y_{g,t-1}$ that is, the delta operator applies to year holding grade constant. Then

$${\Delta y}_{5,t}={\Delta c}_{3,t-2}+{\Delta l}_{4,t-1}+{\Delta l}_{5,t}+{\Delta \varepsilon }_{5,t}$$

That is, changes in measured learning levels for grade 5 from year t-1 to year t can be broken up into changes in learning levels for grade 5 from t-3 to t-2, changes in learning gains for this cohort of students in years t-1 and t, and changes in the noise term. We can think of ${\text{Δ}c}_{3,t-2}$ as the change due to differences between cohorts and ${\text{Δ}l}_{4,t-1}+{\text{Δ}l}_{5,t}$ as the change due to changes in learning trajectories.

Define $L\left({\text{Δ}c}_{5,t},{\text{Δ}c}_{3,t-2}\right)=\gamma {\text{Δ}c}_{3,t-2}$ to be the linear projection of ${\text{Δ}c}_{5,t}$ on ${\text{Δ}c}_{3,t-2}$. Note that if the change due to differences between cohorts is much larger than the change due to changes in learning trajectories, i.e. $\frac{{\text{Δ}l}_{4,t-1}+{\text{Δ}l}_{5,t}}{{\text{Δ}c}_{3,t-2}}\approx 0$, then γ≈1.

Similarly define $L\left({\text{Δ}y}_{5,t},{\text{Δ}y}_{3,t-2}\right)=L\left({\text{Δ}c}_{5,t}+{\text{Δ}\varepsilon }_{5,t},{\text{Δ}c}_{3,t-2}+{\text{Δ}\varepsilon }_{3,t-2}\right)=\beta {\text{Δ}y}_{3,t-2}\;$ to be the linear projection of ${\text{Δ}y}_{5,t}$ on ${\text{Δ}y}_{3,t-2}$. If the delta error terms, ${\text{Δ}\varepsilon }_{5,t}$ and ${\text{Δ}\varepsilon }_{3,t-2}$ are uncorrelated with each other and also uncorrelated with ${\text{Δ}c}_{3,t-2}$, then:

$$plim\left(\beta \right)=\gamma \lambda =\gamma \left(\frac{\sigma_{\Delta c}^{2}}{\sigma_{\Delta c}^{2}+\sigma_{\Delta \varepsilon }^{2}}\right)$$

See Bound et al. (2001) for details. In other words, the coefficient from a regression of ${\text{Δ}y}_{5,t}$ on ${\text{Δ}y}_{3,t-2}$ yields a term which is the product of the linear projection of ${\text{Δ}c}_{5,t}$ on ${\text{Δ}c}_{3,t-2}$ and λ, the ratio of the variance in true changes in learning levels to the variance in measured changes in learning levels.

Thus, if β≈0, either a large share of the overall variance in measured changes is noise (λ≈0) or else cohort effects are small compared to changes in learning and negatively correlated with learning gains (γ≈0).

## Appendix C

Table C1 below presents results from the analysis of ASER reliability using both the correlation decay method (our favored method) and the correlation in deltas method (our alternate method). Note that estimates of the share of variance due to sampling is the same across both methods.

## Footnotes

1 According to the new National Education Policy, which was adopted recently, the centre will create a new National Assessment Centre which will administer the NAS going forward. See https://www.mhrd.gov.in/sites/upload_files/mhrd/files/NEP_Final_English.pdf.

2 In a separate follow-up survey, NAS was also administered to 10th class students.

3 Dubeck and Gove (2015) provide a comprehensive description of EGRA, including some of its limitations.

4 Other indicators for the SEQI come from the Unified District Information System for Education (UDISE). More information about the index can be found at: https://niti.gov.in/content/school-education-quality-index.

5 For more details, see http://www.asercentre.org/Survey/Basic/Pack/Sampling/History/p/Overview/Basic/Pack/History/etc/p/56.html.

6 See page 37 of the NAS operational guidelines for more information on random selection of sections and students.

7 Details of the NAS sample size vary somewhat by source. According to the forward of the NAS district report card report, 120,000 schools and 2.2 million students were sampled. (See http://www.ncert.nic.in/programmes/NAS/pdf/DRC_report.pdf). But according to the initial press release on the NAS, 1,100,000 schools and 2.5 million students were sampled. (See https://pib.gov.in/newsite/printrelease.aspx?relid=173462).

8 For more information on the sampling strategy see https://ihds.umd.edu/sites/default/files/publications/papers/technical%20paper%201.pdf.

9 We include students in grades 2 through 4 in the IHDS sample as otherwise sample sizes per state would be prohibitively small.

10 The official NAS report implies that the state-level class 3 language figures represent the share of students who have achieved both of these learning outcomes. Yet figures for each of the learning outcomes separately and overall class 3 language are virtually identical implying that the overall class 3 score represents the average of the share of students who have achieved each learning outcome.

11 This correlation may also equal 0 under other conditions as well. For example, this may be true if autocorrelation between $u_{t}$ and $u_{t-2}$ is exactly equal to the the negative of the autocorrelation between $\left(\varepsilon_{t}-\varepsilon_{t-1}\right)$ and $\left(\varepsilon_{t-2}-\varepsilon_{t-3}\right)$.

12 These calculations assume that the two components are normally distributed though the results seem to hold for a variety of other distributions as well (including the β and t).
